# Poxvirus-based active immunotherapy synergizes with immune checkpoint inhibitors to cause tumor regression and extend survival in preclinical models of cancer

**DOI:** 10.1186/2051-1426-2-S3-P124

**Published:** 2014-11-06

**Authors:** Susan P Foy, Ryan B Rountree, Joseph Cote, Tracy dela Cruz, Evan Gordon, Erica Trent, Alex Franzusoff, James B Breitmeyer, Stefanie J Mandl

**Affiliations:** 1 Bavarian Nordic, Inc., USA

## 

Combining poxvirus-based immunotherapies which "step on the gas" to activate tumor antigen-specific T cell immune responses with immune checkpoint inhibitors (ICIs) which "release the brakes" on the immune system is a promising direction for enhancing cancer immunotherapy. Evidence for the potential clinical benefit from combination immunotherapy was obtained in a Phase I dose escalation trial. Cohorts of prostate cancer (mCRPC) patients were treated with a fixed dose of PROSTVAC, a poxvirus-based active immunotherapy, plus escalating doses of Ipilimumab, an anti-CTLA-4 ICI. The median overall survival (mOS) of 31.6 months [1] from the combined cohorts was notably longer than the mOS of mCRPC patients from an independent randomized Phase II study (PROSTVAC alone 25.1 months versus placebo 16.6 months; the most pronounced survival benefit (8.5 months) in mCRPC to date) [2]. This potentially synergistic combination of PROSTVAC and Ipilimumab warrants further exploration.

We modeled the benefit of combining poxvirus-based immunotherapy with CTLA-4 blockade using MVA-BN-HER2, which is being developed for breast cancer. In preclinical studies a dramatic increase in mOS was observed in a therapeutic CT26-HER2 lung metastasis model when mice were treated with MVA-BN-HER2 plus CTLA-4 blockade compared to either treatment alone. The improved survival with the combination therapy was accompanied by a striking increase in the magnitude and functional quality of tumor infiltrating HER-2 specific CD8 T cells [3].

Additional ICIs are identified in our preclinical studies as promising candidates for combining with poxvirus-based immunotherapies. Immune checkpoint protein expression is normally induced on activated T cells to regulate activity, and we found that MVA-BN-HER2 treatment resulted in activated CD8 T cells and elevated expression of PD-1, TIM-3, or ICOS. However, immune checkpoint proteins are chronically elevated on exhausted T cells in an immunosuppressive tumor microenvironment. In untreated tumor-bearing mice, a potentially exhausted T cell phenotype was found on CD4 and CD8 T cells characterized by increased expression or co-expression of PD-1, TIM-3, and LAG-3. We compared treatment of MVA-BN-HER2 alone or in combination with ICI antibodies against these immune checkpoint molecules in solid or metastatic CT26-HER2 tumor models. Improved survival was observed with several different combinations, and synergistic efficacy was indicated using the Chou-Talalay method [4]. These studies provide data and rationale for combining poxvirus-based immunotherapies with a variety of ICIs in the clinic.
